# Quantum-Centric Alchemical Free Energy Calculations

**DOI:** 10.1021/acs.jctc.6c00526

**Published:** 2026-06-15

**Authors:** Milana Bazayeva, Zhen Li, Danil Kaliakin, Fangchun Liang, Akhil Shajan, Susanta Das, Kenneth M. Merz

**Affiliations:** † Center for Computational Life Sciences, Lerner Research Institute, 2569 The Cleveland Clinic, Cleveland, Ohio 44106, United States; ‡ Department of Chemistry, 3078 Michigan State University, East Lansing, Michigan 48824, United States

## Abstract

In this work, we extended the book-ending framework with a hybrid quantum-classical workflow that incorporates configuration interaction (CI) calculations into alchemical free energy (AFE) predictions. In the book-ending approach, the Multistate Bennett Acceptance Ratio (MBAR) is applied along a coupling parameter λ to interpolate the system from molecular mechanics (MM) (λ = 0) to a quantum mechanics (QM) (λ = 1) description, and the resulting correction is added to the classically computed AFE. Building on the standard book-ending workflow, we developed an interface that introduces the CI contribution through two backends: (I) a classical PySCF-based backend; (II) a quantum-centric sample-based quantum diagonalization (SQD) method and its extended version (ext-SQD). This latter approach combines real quantum processing units (QPUs) with classical postprocessing to obtain CI energies and gradients. To validate the proposed infrastructure, we computed the book-ending corrections for the hydration free energies (HFEs) of three small organic molecules: ammonia, methane, and water. These benchmarks demonstrate that the CI-level electronic structure calculations, particularly those performed on a quantum hardware, can be naturally incorporated into AFE workflows. Specifically, the CI-corrected HFEs are in reasonable agreement with experimental values, supporting the feasibility of QPU-accelerated free energy predictions. As quantum devices continue to improve in scale and fidelity, they might offer a practical and scalable route to CI-quality electronic-structure data for systems that are challenging for classical approaches. Integrating these CI energies directly into QM/MM simulations could improve the accuracy of free energy methods for systems where electronic correlation plays a significant role, with potential relevance to large biomolecular systems, enhancing our ability to model molecular recognition, enzyme catalysis, and drug–receptor interactions.

## Graphical abstract

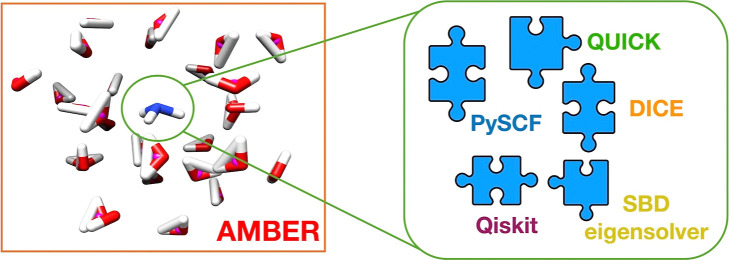

## Introduction

Accurate prediction of molecular binding affinities is pivotal in drug discovery. Among all the available computational strategies, alchemical free energy (AFE) calculations^1^ stand out as a powerful method to estimate key molecular properties, such as hydration free energies (HFE) and ligand–receptor binding affinities.^2–5^ However, the accuracy of current AFE approaches remains limited by the approximations inherent in classical computational methods, particularly the selection and parametrization of force fields (FFs). This is becoming more evident as drug discovery extends into more complex environments involving metal ions, nucleotides, and interacting molecules.^6–8^

Classical approaches do not include subtle electronic contributions and polarization effects that occur in such complex systems.^9,10^ Recent advances in quantum chemistry have significantly impacted drug discovery and free energy calculations, offering promising avenues for improving accuracy through QM/MM methodologies.^10–14^ However, direct QM/MM free energy calculations remain challenging due to the computational demand of the QM treatment arising from the need for extensive configurational sampling.^15–17^

In light of this, the book-ending approach represents an effective alternative for overcoming the limitations of the full QM/MM AFE calculations. In this indirect approach, the free energy is initially computed leveraging the classical FFs, and QM/MM accuracy is later introduced by applying the end-state corrections. For each state, the free energy difference between the MM and QM/MM descriptions is computed and combined with the classical result. In this workflow, the chemical composition remains unchanged throughout the MM → QM/MM transformation, significantly reducing the required sampling.^15^ Building on the work of Gao^18,19^ and Warshel^20^ on the use of reference potentials, and further developed by Woodcock, Boresch, König, Brooks.,^21–29^ Giese et al. proposed the book-ending approach for FF parametrization, in which the parameters are optimized to reproduce QM/MM forces.^30^ A further development improves the accuracy using a machine learning workflow.^15^ Nevertheless, the predicted free energies still deviate from the experimental data, highlighting the importance of employing higher-level ab initio methods, as explored in the present work. Density functional theory (DFT) is often used as a reference method for its trade-off between computational cost and performance, but it can fail in describing systems with high accuracy.^31^

In contrast, the full configuration interaction (FCI) method provides the exact solution of the Schrödinger equation within the chosen basis set and is commonly used as a benchmark for less accurate quantum techniques.^32,33^ The prohibitive computational cost of FCI creates the need for approximate methods, such as the selected CI (SCI) approach, which retains only the most significant determinants to reach the near-FCI level of accuracy with a much more favorable computational expense.^32,34–36^

Beyond SCI, other classical approaches target near-FCI level of accuracy in larger active spaces. Density matrix renormalization group (DMRG),^37^ full configuration interaction quantum Monte Carlo (FCIQMC),^38^ and auxiliary-field quantum Monte Carlo (AFQMC)^39^ represent the current classical state of the art for approximating the FCI limit. Despite considerable progress in the classical CI field, the scaling problem related to the system size continues to motivate the exploration of other strategies.

Within the realm of SCI methods, a scalable quantum approach is offered by sample-based quantum diagonalization (SQD) method, combined with its extended version (ext-SQD).^40–45^ This approach provides a pathway toward near-FCI, enabling simulations of systems that remain challenging for classical CI techniques. Ext-SQD, in particular, enhances SQD by improving the efficiency and stability of the sampling process, thereby facilitating accurate treatments of larger systems with strong electronic correlation.^43,45^ SQD is a sample-based approach suitable for the currently available NISQ devices. Quantum phase estimation (QPE) is anticipated to be a promising quantum algorithm with a wide applications range, chemistry included. At present, QPE suffers of limitations in terms of qubit number, circuit depth, and coherence time postponing its use to the fault-tolerant era.^46,47^ The interface presented in this work is flexible by design and can be extended to incorporate new algorithms, such as QPE, as quantum computing progresses.

Recent works have demonstrated the application of quantum computing to real study cases, such as drug discovery, enzymatic reactions, and protein–ligand interactions.^48–50^ First efforts to include quantum computing in free energy calculations have also emerged,^51,52^ but a direct integration of such hardware into free energy workflows remains unexplored.

To enable efficient coupling between the quantum processing unit (QPU) and classical CPUs, we developed a dedicated interface that also supports conventional CI simulations, allowing direct benchmarking of quantum-centric workflows. Our CI- and SQD/ext-SQD-based book-ending framework can be further expanded through density matrix embedding theory (DMET),^53^ and recent success under implicit solvation motivates its extension to AFE calculations within explicit solvent.^41,54^

In this work, we test the CI-corrected book-ending scheme on a set of small molecules^55,56^ to establish a proof-of-concept validation. This implementation represents a step toward practical, quantum-augmented free energy simulations, with the longer-term goal of tackling the strong electronic correlations found in drug–receptor interactions and enzyme catalysis.

## Methods and Computational Details

### Structure Preparation and MM HFE Calculation

The initial coordinates for ammonia, methane, and water were generated using the LEaP module of the AMBER24 software package.^57^ Ammonia and methane were parametrized using the General AMBER Force Field (GAFF2),^58^ with atom types and bonded parameters automatically assigned by LEaP. Atomic partial charges for these molecules were derived using the Restrained Electrostatic Potential (RESP) fitting method,^59^ based on molecular electrostatic potentials calculated at the B3LYP level with the 6-31G* basis set using the Gaussian software package.^60^ Water was modeled directly using OPC3.^61^ Each system also included the corresponding dummy molecule required for the alchemical transformation in the HFE protocol. All solvated systems were embedded in cubic OPC3 water box with 24 Å of padding around the solute, and periodic boundary conditions (PBC) were applied to eliminate edge effects and mimic the bulk solvent environment. All systems were first relaxed using a two-stage energy minimization protocol consisting of 10,000 steps of steepest descent followed by 10,000 steps of conjugate gradient. Long-range electrostatics were evaluated with the Particle Mesh Ewald method^62^ employing a 10 Å real-space cutoff and a 48 × 48 × 48 FFT grid. Each system was then equilibrated in two phases. In the NVT phase (360 ps), the temperature was gradually raised from 0 to 300 K in 50 K increments using Langevin dynamics with a collision frequency of 2 ps^–1^. This ensured a stable and efficient thermal regulation during the heating phase. This was followed by a 300 ps NPT equilibration at 300 K and 1 bar using the Berendsen barostat.^63^ Thermodynamic integration (TI) was used throughout for the free energy calculations.

The equilibrated structures provided the initial configurations for the classical HFE calculations as well as for the book-ending corrections. The HFE of each solute was computed using a seven-window TI scheme. In this approach, the hybrid potential for each λ window is defined as

1 $$U\left(\lambda \right)=\left(1-\lambda \right)U_{0}+\lambda U_{1}$$

where *U*(λ) denotes the system energy at coupling parameter λ, while *U*_0_ and *U*_1_ correspond to the energies of the fully solvated solute (λ = 0), and the energy of the dummy solute state (λ = 1). The Helmholtz free energy difference between these two states is given by

2 $$\Delta A=\int_{0}^{1}\left\langle \frac{dU}{d\lambda }\right\rangle d\lambda$$

which was evaluated using a seven-point Gaussian quadrature

3 $$\Delta A=\sum_{i=1}^{7}\left(c_{i}\times \left\langle \frac{dU_{i}}{d\lambda_{i}}\right\rangle \right)$$

in eq 3, *c*_*i*_ are the Gaussian quadrature weights {0.065, 0.140, 0.191, 0.209, 0.191, 0.140, 0.065}, and the corresponding abscissae are λ_*i*_ {0.025, 0.130, 0.297, 0.500, 0.703, 0.870, 0.975}. Each λ-window was simulated for 3 ns under NVT conditions, giving a total TI simulation time of 21 ns. The resulting Helmholtz free energy is reported here as Gibbs free energy.^64^ All HFE calculations were performed in triplicates.

### Book-Ending Simulations

As mentioned in the introduction, the book-ending method is an indirect approach to achieve QM accuracy in AFE calculations without performing a full QM/MM simulation throughout the entire alchemical pathway. Here, the classical MM simulations are paired with a correction that is evaluated only at the alchemical end-states (Figure 1). The correction is obtained by smoothly transitioning the system from a purely MM potential (λ = 0) to a QM/MM description (λ = 1) using a series of intermediate states, during which only the potential energy function is modified while the atomic configurations remain unchanged. In line with the original book-ending protocol by Giese et al.,^15,16^ our workflow employs the Multistate Bennett Acceptance Ratio (MBAR) to compute the MM → QM/MM book-ending free energy corrections. MBAR combines the potential energies sampled across the six alchemical λ states (0.00, 0.20, 0.40, 0.60, 0.80, 1.00) to obtain statistically optimal free energy differences. The three solutes (ammonia, methane, and water) were modeled similarly to the protocol described for the classical HFE calculations. For each molecule, simulations were performed over six λ windows, with each window including 1 ps of equilibration and 1 ps of production in the NVT ensemble under PBC, yielding 6 ps of production data in total per simulation. Three independent replicates were performed for each system for statistical robustness. The data set of each trial was combined in a single MBAR analysis rather than averaged, yielding the final correction and its statistical uncertainty.

**Figure 1:**
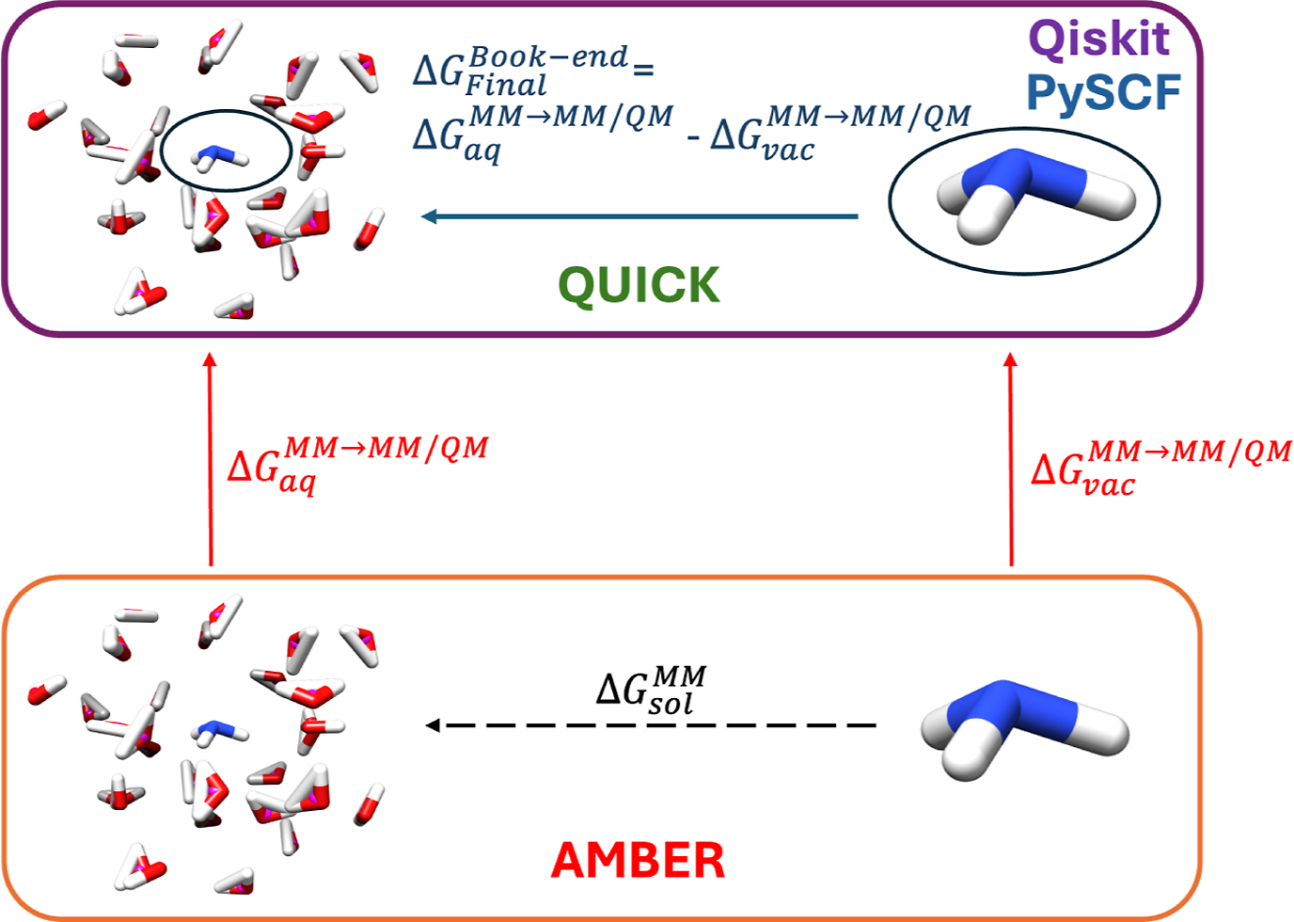
Schematic representation of the book-ending energy correction procedure. At the bottom, the HFE is computed using classical MM force fields (dashed black arrow). The book-ending approach employs thermodynamic integration (TI) to switch the potential from MM to QM/MM at each end-state (vertical red arrows). The quantum contribution can be calculated using one of three approaches: HF as implemented in the QUICK package, HCI within PySCF framework, or the quantum-centric SQD/ext-SQD approach. The free energy difference obtained from TI is analyzed with MBAR to yield the quantum correction (blue arrow), which is then added on top of the classical HFE.

Although the simulations are relatively short by modern standards, the book-ending method does not require a full TI between states. Instead, it evaluates the energy difference between the MM and QM Hamiltonians for the same molecular state without involving nonphysical intermediates. Given that our benchmark consists of small molecules with a single heteroatom, the MM and QM descriptions remain closely related; this leads to substantial phase-space overlap, allowing for reliable corrections even on these time scales.

As the field evolves and computational resources grow, longer simulation time scales will become increasingly accessible. More complex systems, in particular, will likely require extended sampling and potentially additional λ windows to accurately bridge the MM → QM/MM energy gap.

During equilibration, each λ window was initialized from the preceding one. In contrast, production runs were initiated independently using the equilibrated structure of each respective window. Langevin dynamics with a collision frequency of 5 ps^–1^ was used to maintain the target temperature of 298 K. No pressure control was applied, and a nonbonded interaction cutoff of 10 Å was used. All bonds involving hydrogen were constrained using SHAKE. The solute, treated as the QM region, was subjected to hydrogen mass repartitioning^65^ to enable a 1 fs the integration time step. Two input files were used for each λ window: one performing a purely classical MM calculation and the other enabling QM/MM with electrostatic embedding. The QM region was described at the Hartree–Fock (HF) level with the 6-31G* basis set, using QUICK as the QM engine.^66,67^ In this work, the heat-bath configuration interaction (HCI) method was employed as a classical reference to benchmark the SQD-based workflow.^68^ The production runs were performed using three different computational setups: (I) HF (only QUICK), (II) HF + CI external solver, (III) HF + SQD/ext-SQD, with the external computation carried out on quantum hardware and refined through the SQD/ext-SQD solver. For statistical analysis, for each setup three independent replicates were performed. External CI solvers were activated only during the production runs every 10 steps and we considered all orbitals within the selected basis set except for the frozen core orbital. For the HCI simulations a subspace expansion cutoff^68^ of ϵ_1_ = 1 · 10^–5^ was employed, and the DICE solver was used for Hamiltonian diagonalization on 24 CPUs. Simulations on real quantum hardware were executed using the Heron chips ibm_cleveland and ibm_marrakesh with 200000 quantum shots. The initial SQD postprocessing was done using 10 batches of 1000 samples each and two iterations. The ext-SQD was applied as a final refinement step to the lowest-energy batch using a threshold of d ’ = 1 · 10^–4^, which corresponds to the weight of the particular configuration in the overall wave function. By applying single excitations to only the most dominant configurations (d’) we ensure the compactness of the resulting subspaces. For the SQD/ext-SQD solver, the selected basis diagonalization (SBD) solver was used to perform the diagonalization on 24 CPUs. SQD and ext-SQD details can be found in the Supporting Information section.

### Multistate Bennett Acceptance Ratio (MBAR) Analysis

In line with the original book-ending protocol by Giese et al.,^15,16^ our workflow employs MBAR to compute the MM → QM/MM book-ending free energy corrections. The MBAR formalism^69^ extends the Bennett Acceptance Ratio to multiple thermodynamic states, providing the free energy differences from the combined ensemble of all states. We start from the Helmholtz free energy as the difference between two thermodynamic states λ_*i*_ and λ_*j*_

4 $$\Delta A_{ij}=-\frac{1}{\beta }\ln\frac{Q_{j}}{Q_{i}}$$

where *Q*_*i*_ and *Q*_*j*_ are the corresponding partition functions defined through the Boltzmann distribution. β = 1/(*k*_*B*_ *T*), with *k*_*B*_ being the Boltzmann constant and *T* the absolute temperature. eq 4 can be written as an identity

5 $$Q_{i}\langle \alpha_{ij}\times e^{-\beta U_{j}\left(r\right)}\rangle_{i}=Q_{j}\langle \alpha_{ij}\times e^{-\beta U_{i}\left(r\right)}\rangle_{j}$$

here, ⟨···⟩ denotes the ensemble average over configurations sampled at state λ_*i*_ (and analogously for λ_*j*_), and α_*ij*_ is a weighting factor whose specific form is chosen to minimize the variance of the free energy estimator. Generalizing to *K* states, α_*ij*_ takes the form

6 $$\alpha_{ij}=\frac{\frac{N_{j}}{{\mathrm{Q̂}}_{j}}}{\sum_{k=1}^{K}\frac{N_{k}e^{-\beta U_{k}\left(r_{jn}\right)}}{{\mathrm{Q̂}}_{k}}}$$

*K* is the total number of λ_*k*_ alchemical states (six in this study, from λ_1_ = 0.00 to λ_6_ = 1.00, in increments of 0.20). *N*_*k*_ is the total number of sampled configurations at state λ_*k*_, and ${\mathrm{Q̂}}_{k}$ is the estimated partition function of the same state. The free energy of each state is related to its partition function by ${Â}_{k}=-\frac{1}{\beta }\ln{\mathrm{Q̂}}_{k}$ . Substituting eq 6 back to eq 5 and finally to eq 4, leads to the MBAR equation

7 $${Â}_{i}=-\frac{1}{\beta }\ln\sum_{j=1}^{K}\sum_{n=1}^{N_{j}}\frac{e^{-\beta U_{i}\left(r_{jn}\right)}}{\sum_{k=1}^{K}N_{k}e^{\beta {Â}_{k}-\beta U_{k}\left(r_{jn}\right)}}$$

Given an initial guess for the set $\left\{\hat{A_{k}\;},k\in \left\{1,2,3,...,6\right\}\right\}$ , the free energy parameters are updated by iteratively solving eq 7. Book-ending simulations were performed in the NVT ensemble and eq 7 yields a Helmholtz free energy to represent the Gibbs free energy correction from MM to QM/MM state.^64^

### Interfacing AMBER with External CI Solvers

To couple classical MD with quantum circuit-based computations, we implemented a modular extension of the sander engine, one of the MD engines in the AMBER suite. The operational workflow of the interface is illustrated in Figure 2. In this framework, QUICK^66,67^ is maintained as the native QM engine for the computation of HF energies and gradients at each MD step. In the present version, QUICK operates through a file-based interface (FBI), which sander uses to invoke the QM calculation, parse the resulting energies and gradients, and integrate them into the QM/MM force evaluation (left side of Figure 2). This same architecture is the base of the extended coupling to external solvers using PySCF.^70,71^ Through a series of Python scripts, PySCF generates the one- and two-electron integrals, constructs the electronic Hamiltonians, and creates a unified backend interface. This extension enables the QM region to be treated using both classical post-HF treatments via the CI solver, and the quantum-centric simulations based on the SQD/ext-SQD framework (right side of Figure 2). For the latter, PySCF was integrated within the Qiskit ecosystem^72,73^ to send quantum circuits to hardware backends and collect the resulting measurements used in the SQD/ext-SQD postprocessing.^40,42–45,74^

**Figure 2:**
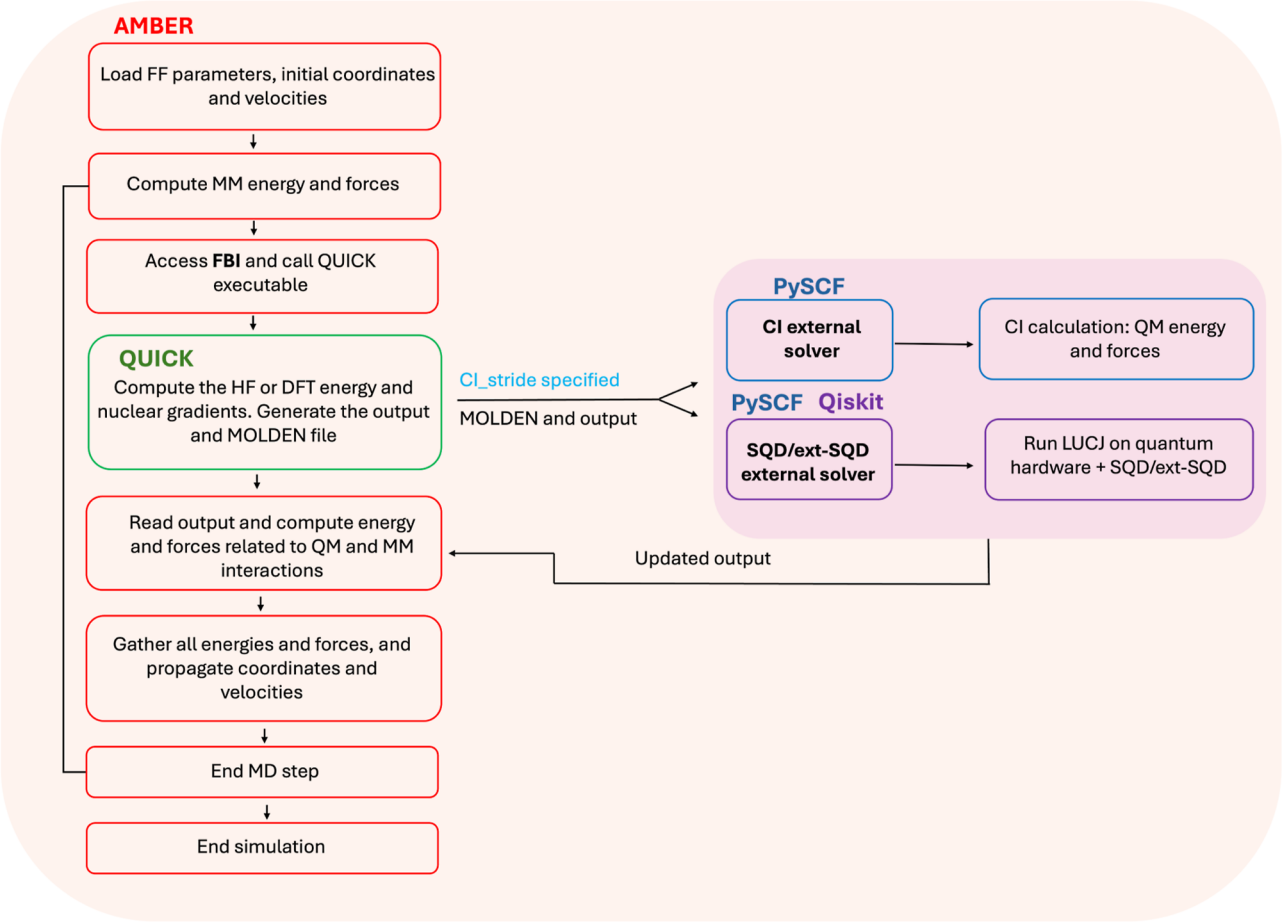
Workflow of the standard sander functionality within the QM/MM scheme (left), extended with the interface presented in this work (right). The steps managed by sander are shown in red, those performed by the QUICK engine in green, and those handled by external CI solvers are enclosed in the purple box. The CI simulations are performed using PySCF (boxes in light blue), while for quantum-centric SQD/ext-SQD calculation leverage a synergistic combination of PySCF and Qiskit (the boxes in purple).

Post-HF calculations are triggered at intervals defined by the CI_stride keyword. At these steps, the workflow branches into one of two external-solver routes: (I) the conventional CI solver, or (II) the SQD/ext-SQD solver, where the CI component is computed by a QPU with the LUCJ Ansatz,^75^ and refined through the Qiskit Addon: SQD + ext-SQD. Notably, in the present work we also enable analytic gradient evaluation within the SQD/ext-SQD framework, allowing the resulting forces to be incorporated back into the MD propagation. This constitutes a key methodological advancement over previous SQD implementations, which were limited to the evaluation of energies alone. On the left-hand side of Figure 2, the standard QUICK-based scheme is shown: at each MD step, sander computes the MM contributions, then uses the FBI to invoke QUICK, which evaluates the HF (or DFT) energy and nuclear gradients for the QM region. These results are reintegrated into the QM/MM force evaluation, allowing sander to propagate the atomic positions and velocities. This procedure repeats at every MD step. On the right-hand side, the extended workflow retains the same basic structure, with QUICK providing HF energies and gradients at each step, but introduces the CI_stride mechanism. At certain MD steps, the QM computation is redirected to an external solver. This redirection leverages the MOLDEN file generated by QUICK, which contains the geometry and HF molecular orbitals required by both the CI and SQD/ext-SQD workflows. The external solver returns energies and gradients, which are formatted according to the FBI conventions and seamlessly reintegrated into the AMBER workflow. Sander then continues the MD propagation using the updated QM/MM forces while preserving the efficiency of the original QUICK-based interface. Refer to the Supporting Information for additional technical details on the development of the interface.

## Results and Discussion

To validate the interface and evaluate its impact on the accuracy of the book-ending corrections, we computed the HFEs of three small molecules (ammonia, methane, and water) and subsequently applied quantum-level corrections. During the initial stages of the interface development, we carried out exploratory tests using the minimal STO-3G basis set, with the QM region treated at the FCI level (reported in the Supporting Information). These preliminary calculations served as diagnostic benchmarks to assess the behavior of the systems and to debug the book-ending workflow involving the external CI solvers. These tests indicated that larger basis sets introduce an unfavorable electron to orbital ratio, which can lead to instabilities in the SQD and ext-SQD approaches. We therefore selected 6-31G* as the minimal basis set that provides a realistic QM description while keeping a reasonable electron-to-orbital ratio. At this level, FCI is no longer feasible, so we switched to the HCI approach,^68^ which offers a favorable balance between accuracy and computational cost and serves as a reference.

Upon switching to 6-31G*, the book-ending corrections for ammonia and water exhibit a systematic shift, while methane remains largely unaffected. As a consequence, the QM-corrected HFEs become overly negative despite the satisfactory performance of the classical baseline. This behavior is attributed to the well-known tendency of HF/6-31G* to over polarize specific chemical groups, leading to enhanced dipole moments in molecules containing heteroatoms or involved in directional hydrogen bonding.^76–78^ To mitigate this imbalance, we reparametrized the Lennard–Jones (LJ) parameters for ammonia and water. The reparametrization was performed by changing the LJ well depth (ε) and radius (σ) over a grid of values (Figures S4 and S5). For each combination, the HFE was recomputed, and the parameters were selected as those that best reproduced the experimental values after book-ending correction. The resulting MM contributions are better matched to the increased QM polarization, leading to more consistent and physically meaningful book-ending corrections (the final HFE values are reported in Table 1).

**Table 1:** HFE Values Obtained With MM Approach and Three Book-Ending Correction Protocols (all Values are in Kcal/Mol)

system	MM HFE	HF protocol	HF + HCI protocol	HF + SQD/ext-SQD protocol	MNSol
ammonia	–1.14 ± 0.22	–4.84 ± 0.16	–4.82 ± 0.34	–4.43 ± 0.18	–4.29
methane	2.56 ± 0.03	2.09 ± 0.04	2.13 ± 0.09	2.16 ± 0.06	2.00
water	–5.54 ± 0.45	–6.43 ± 0.39	–6.32 ± 0.33	–6.38 ± 0.26	–6.31

Table 1 presents a comprehensive overview of the results computed with the three different protocols (HF, HF + HCI, and HF + SQD/ext-SQD, see Methods). Here, we report the MM-calculated HFEs after LJ reparametrization, the corresponding book-ending corrected values, and the Minnesota Solvation Database (MNSol) values.^79^

We first address the results for ammonia and water, as these polar molecules represent the most challenging systems for the proposed book-ending strategy. The HFE of ammonia is −1.14 kcal/mol. The HF and HF + HCI protocols yield almost identical final values, with a deviation of ∼0.53 kcal/mol from the MNSol reference. The protocol involving the CI contribution generated from quantum hardware shows a minimal difference of 0.14 kcal/mol from the reference value. Water has a HFE value of −5.54 kcal/mol, already within 1 kcal/mol from the reference value of −6.31 kcal/mol. The best agreement is obtained with the HF + HCI protocol, which reproduces the reference HFE almost exactly, followed by the SQD/ext-SQD protocol (0.07 kcal/mol difference). The standard HF calculations yield a deviation of approximately 0.12 kcal/mol from the MNSol value. For methane, the only nonpolar molecule in our benchmark, the MM prediction (2.56 kcal/mol) is already in good agreement with the MNSol database (2.00 kcal/mol). The HF protocol introduces a correction of −0.47 kcal/mol (Table S4, Supporting Information), with a final value of 2.09 kcal/mol. The inclusion of the CI contribution through HF + HCI and HF + SQD/ext-SQD protocols yields final values of 2.13 and 2.16 kcal/mol, respectively. For this system all the quantum protocols converged toward the MNSol reference value without adjusting the standard LJ parameters. Altogether, these results indicate that, once the LJ parameters are reoptimized to account for the enhanced QM polarization, the book-ending corrections lead to accurate and physically consistent HFEs.

All the applied protocols show comparable performances with the difference between HF, HF + HCI, and HF + SQD/ext-SQD within the statistical uncertainties. For our systems, HF alone was able to reproduce the experimental reference reasonably well, while the CI-enhanced results did not show systematic improvement over HF. However, these findings validate the newly developed interface and demonstrate that post-HF corrections obtained from quantum hardware can be meaningfully embedded into QM/MM free energy calculations within the AMBER package. We show that the HF + SQD/ext-SQD protocol yields free energy estimates consistent with those obtained with the HF + HCI reference and with the experimental data, despite relying on CI information generated on quantum hardware and incorporated only intermittently along the MD trajectory. This establishes a proof of principle that quantum hardware-derived CI information can be used to refine the AFE calculations within the classical MD framework. Improvements of the book-ending corrections are expected when strongly correlated systems are investigated, allowing a larger fraction of post-HF information to be incorporated into the free energy estimate. The dependence of the corrections on the CI evaluation (stride) remains open for future investigations. Overall, the book-ending framework provides a practical and scalable route for integrating near-FCI quantum hardware into MD simulation workflows, and this work represents the first embedding of such hardware within an alchemical free energy scheme with both energy and gradient evaluation.

## Conclusions and Outlook

AFE methods are widely used to estimate free energy differences associated with molecular transformations, a key requirement for predicting properties such as binding affinity. Although quantum algorithms have been extensively studied for electronic structure calculations, their application to AFE transformations has so far been limited. In this work, we address this largely unexplored problem, investigating quantum algorithms tailored for free energy estimation as a promising route for deploying quantum hardware to chemistry-related challenges relevant to applications such as drug discovery.^2,8^ A central contribution is the integration of CI methods, HCI and SQD/ext-SQD, within established classical AFE workflows, extending beyond the traditional book-ending corrections that have so far been limited to HF and DFT approaches. Notably, this study is the first, to the best of our knowledge, to compute nuclear gradients using the SQD method; previous SQD investigations were restricted to total energy evaluations alone.^40,74^

The proposed workflow further bridges the molecular dynamics package AMBER with quantum backends capable of CI simulations, enabling seamless hybrid quantum-classical free energy calculations. This integration not only enables both conventional simulations on classical hardware and quantum-centric SQD simulations on quantum devices, but also highlights the synergy between the classical and quantum platforms. Our approach opens the door to the systematic inclusion of CI calculations in the free energy framework, as well as to the validation and benchmarking of current databases.^79^ Our tests on small molecules demonstrate that CI-based book-ending corrections obtained from quantum-centric methods can be successfully incorporated into the AFE workflow, yielding estimates consistent with MNSol reference values. These results are encouraging and support the feasibility of quantum-centric AFE workflows as quantum algorithms and devices continue to mature. For applications such as drug discovery, the book-ending approach keeps the QM evaluation at the end-states only, making CI-level corrections computationally tractable. Large-scale screening, however, will require further advances in quantum hardware and postprocessing. Moreover, free energy perturbation schemes are naturally well suited for parallel execution across multiple quantum devices, since the λ windows of the production phase are independent by design. Importantly, parallelism will facilitate the use of longer free energy simulations, which will improve convergence and overall accuracy of the calculations over the shorter simulation time scales used herein. The practical exploitation of this parallelism is currently constrained by classical postprocessing overhead. This limitation arises from the cost of the SQD/ext-SQD postprocessing required at each QPU-delegated step, which dominates the wall-clock time. Although the SBD solver allows the batch diagonalizations to be run in parallel across multiple CPUs, this step remains the bottleneck. This inherent structure nonetheless provides a clear pathway toward scalable and efficient AFE workflows as classical and quantum resources coevolve, particularly in preparation for the post-NISQ era.

## Supplementary Material



## Notes

Technical details of the QUICK interface with the external solvers. Details of the quantum-centric HCI and SQD/ext-SQD simulations. Book-ending benchmark with STO-3G and the corrections computed for the 6-31G* for the three molecules in the benchmark. Lennard-Jones reparametrization grids for ammonia and water. List of the abbreviations used in the work (PDF)

## Notes

List of the GitHuB repositories of the codes used in the present work. Book-ending Tutorial: https://ambertutorials-rutgerslbsr-c744272d5a9c1169e0dc9e19b8d800019105.gitlab.io/workshop/06_mlpBookending_gwTP/mm2qmmm/index.html/#mjx-eqn\%3AE\%3ABEaq. Qiskit and Qiskit IBM Runtime: https://github.com/Qiskit/qiskit, https://github.com/Qiskit/qiskit-ibm-runtime. ffsim: https://github.com/qiskit-community/ffsim. Qiskit SQD addon: https://github.com/Qiskit/qiskit-addon-sqd. DICE solver: https://github.com/sanshar/Dice. SBD solver: https://github.com/r-ccs-cms/sbd. PySCF: https://github.com/pyscf/pyscf. PyCI: https://github.com/theochem/PyCI. The tutorial demonstrating full SQD workflow is available below: https://qiskit.github.io/qiskit-addon-sqd/tutorials/01_chemistry_hamiltonian.html.
