# Supplementary material for: Quantum-Centric Alchemical Free Energy Calculations
Source: J Chem Theory Comput. 2026 Jun 15;22(13):6835–43. doi: 10.1021/acs.jctc.6c00526 (PMC13374019; doi:10.1021/acs.jctc.6c00526)
Supplement: Supplementary file 1 [file ct6c00526_si_001.pdf]

Quantum-Centric Alchemical Free Energy Calculations

Milana Bazayeva,† Zhen Li,† Danil Kaliakin,† Fangchun Liang,† Akhil Shajan,† Susanta Das,† and Kenneth M. Merz Jr.*,†

†*Center for Computational Life Sciences, Lerner Research Institute, The Cleveland Clinic, Cleveland, Ohio 44106, United States*

‡*Department of Chemistry, Michigan State University, East Lansing, Michigan 48824, United States*

E-mail: kmerz1@gmail.com

QUICK interface with PySCF and Qiskit Addon: SQD/ext-SQD

To enable the inclusion of CI-level electronic structure information within QM/MM trajectories performed on classical hardware, we extended the *sander–QUICK* file-based interface (FBI).[1,2] This framework allows CI calculations to be embedded directly into standard AMBER simulations.[3] Two options are supported: (I) an CI solver,[4] accessed through the PySCF 2.8.0[5,6] interface with DICE, and (II) the SQD/ext-SQD module, which builds on the PySCF infrastructure and employs Qiskit[7,8] to connect to a real quantum processor and generate electron configurations via the LUCJ ansatz, followed by SQD/ext-SQD post-processing. The PySCF code is responsible of the data exchange between the classical simulations and the external solvers. Its integration within the AMBER Fortran module qm2_extern_quick_module.F90 allowed the creation of the interface shown in Figure S1. The redirection frequency (in MD steps) of the QM calculations to the ex-

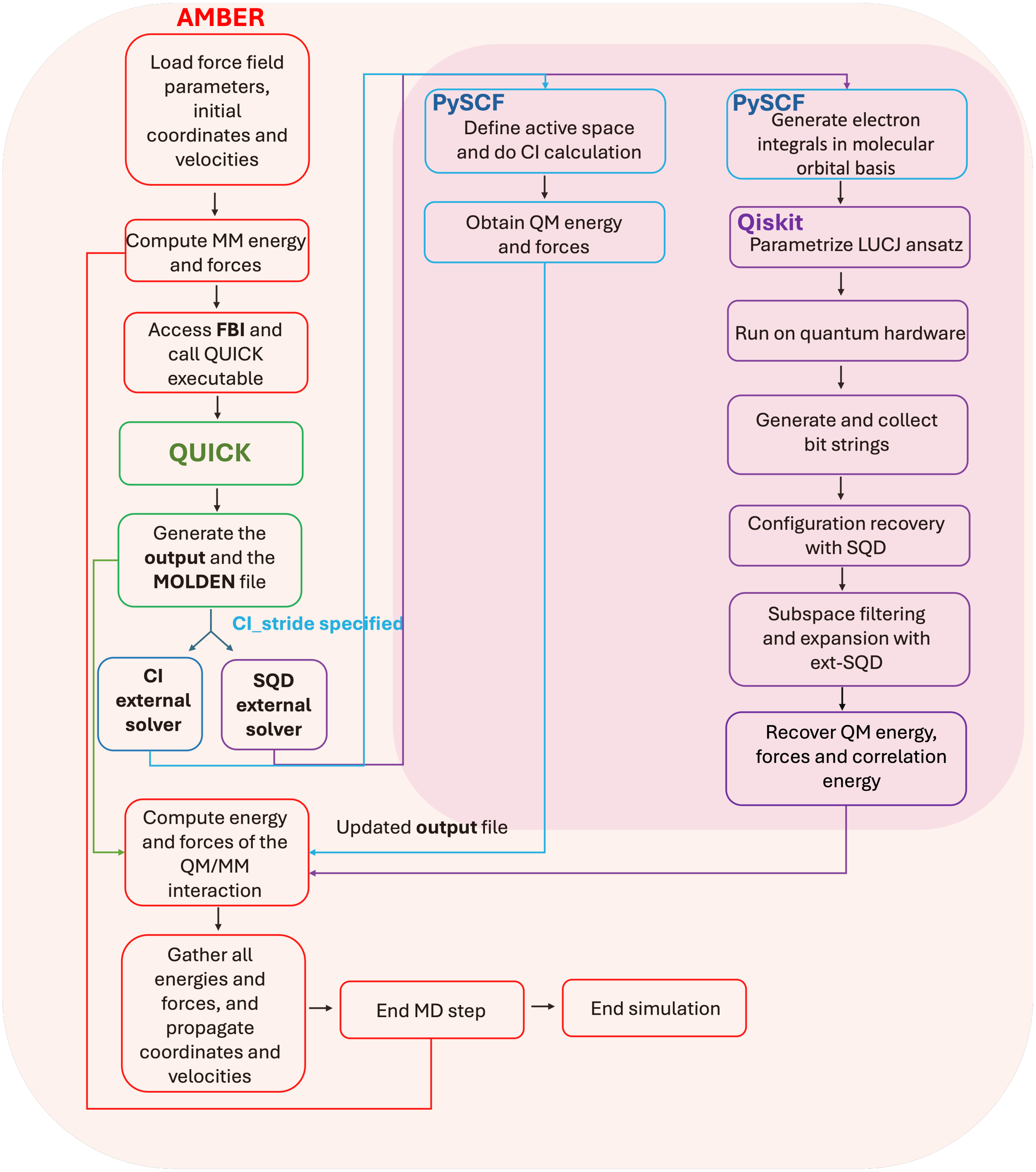

Figure S1: Schematic representation of the integration of AMBER's sander module, the QUICK quantum engine, and external CI solvers. The CI_stride keyword controls the redirection of the QM computation to one of the two external CI solvers. The color scheme highlights the workflow: steps managed by sander are shown in red, those performed by QUICK engine in green. The purple box represents the steps performed by either the HCI calculations, or by quantum-centric SQD/ext-SQD workflow. PySCF code is reported in light blue, whereas the steps controlled by Qiskit are in purple.

ternal solvers is managed by the dedicated keyword: `CI_stride`. QUICK's FBI mode supports custom input template to define the QM method, basis set, and target measures to compute such as energy and gradient. This template also allows the generation of a MOLDEN file, which contains the molecular orbital and geometry data essential for both the CI and SQD/ext-SQD solver. Shared functionalities are implemented in the `quick_parsing_utilities.py` script. This script parses the `QUICK_job.out` file, extracts the coordinates and any associated MM point charges, forwards them to the external solver, and finally integrates the computed energy, nuclear gradient and correlation energy into a new output file, `QUICK_job_PySCF_modified.out`. Depending on the simulation environment (either in aqueous solution or in vacuum) the system is initialized differently. In the gas-phase simulations, with no external charges, and a standard HF is performed. When the system is solvated, the coordinates and charges of the water molecules are defined within PySCF and the QM/MM module is used, enabling electrostatic embedding in the MM environment. This steps creates the HF object that is populated with orbital coefficients, energies, and occupations extracted from the MOLDEN file.

HCI Calculations

The `PySCF-run.py` script is the main driver of the HCI workflow. It loads the molecular orbitals and geometry from the MOLDEN file, reconstructs HF object, and defines the active space using either AVAS procedure or a frozen-core option specified in the `config.yaml` file. This configuration file also specifies the parameters for the HCI calculation (HCI thresholds, the maximum number of Davidson iterations) and the SLURM resource settings required for the batch calculations. SLURM job submission and queue monitoring are handled through the helper functions defined in `submission_utilities.py`, which submit the HCI batch job, extract the SLURM job ID, and block further execution until the external CI calculation has completed. Once the HF reference and the active space are computed, `PySCF-run.py` generates a batch-specific SLURM submission script and initiates the HCI

computation. All scripts required for the CI workflow are contained in the `Workflow` directory and are here described as follow: I) `solve_from_quick_HCI.py`, the core CI solver integrating PySCF and DICE. Here, is defined the `solve_from_quick_HCI()` function and the customized CASCI kernel that replaces the standard CASCI procedure in PySCF. The modified kernel constructs the active-space Hamiltonian and delegates its diagonalization to the DICE SCI solver, accessed via the Qiskit addon interface. The interaction with the DICE solver (qiskit-addon-dice-solver version 0.3.0) is mediated through PySCF's Selected-CI interface, specifically via `fci.selected_ci.SelectedCI()`solver object, which serves as the wrapper for the external CI computation. II) `solver_HCI.py`, the batch execution script, is called by PySCF-run.py via SLURM submission. It reconstructs the HF object and the active space for the current batch, prepares a temporary working directory, and then invokes the routine `solve_from_quick_HCI()`. The CI diagonalization itself is run as a SLURM batch calculation using the submission template `submit_batch_template_HCI.sh`, which is instantiated by `PySCF-run.py` before job submission. Upon completion of the DICE diagonalization, `solve_from_quick_HCI()` returns the HCI energy, CI amplitudes, spin-resolved orbital occupations, and the analytic nuclear gradient. These quantities are saved to a compressed `.npz` archive in the `Workflow` directory. `PySCF-run.py` then retrieves this archive, extracts the CI energy and gradient, and incorporates them into a modified QUICK output file (`QUICK_job_PySCF_modified.out`), replacing the HF value used by *sander* during subsequent QM/MM propagation.

SQD/ext-SQD Calculations

The SQD solver workflow starts with the `LUCJ-run.py` that prepares the quantum input by saving the one-electron and two-electron integrals of the orbitals within the active space. These integrals are written in a FCIDUMP file together with the nuclear repulsion and the electron number. In the following step the FCIDUMP is read back to run a Coupled Cluster Singles and Doubles (CCSD) calculation, from which the single t_1 and double t_2 excitation

amplitudes are extracted. We generate the LUCJ circuits using the ffsim library (version 0.0.54)[9] interfaced with Qiskit 2.0.0.[7,8] The t_2 amplitudes are used to parametrize the LUCJ ansatz, constructed via the `UCJOpSpinBalanced` class and with a zig-zag shape, as shown in Figure S2. The circuits include 1 repetition (`n_reps=1`) and entangling connections defined by nearest-neighbor and on-site interaction patterns in the active space. Circuit transpilation is performed with optimization level 3 and includes a custom pre-initialization step provided by ffsim. Quantum error mitigation is applied through gate twirling (while measurement twirling is disabled), as enabled by the `SamplerV2` primitive in Qiskit's runtime library (version 0.43.1). In Figure S2, we show an example of circuit layout for each molecule, and more details about the circuits are reported in Figure S3.

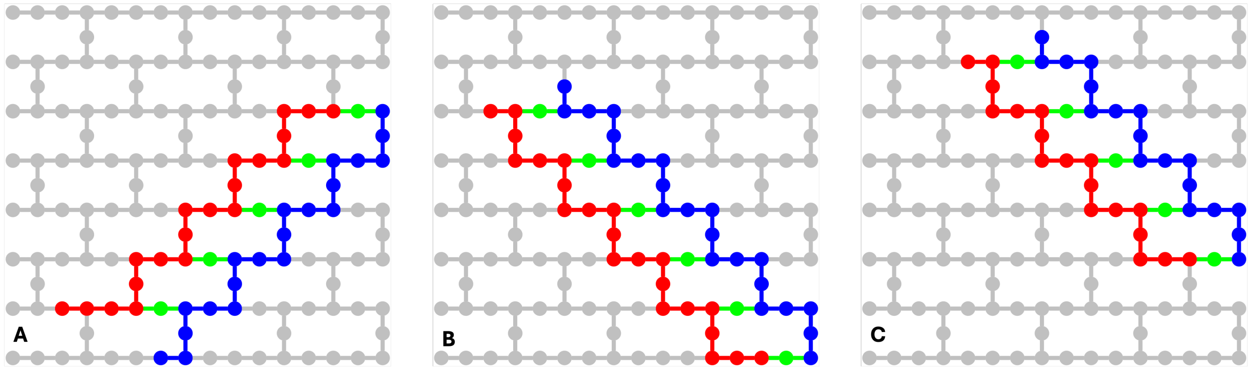

Figure S2: Qubits layout of the LUCJ circuit for each of three solutes investigated in the present study. A) (10e,20o) ammonia, B) (10e,22o) methane and, C) (10e,18o) water. Qubits corresponding to the occupation numbers of α and β spin-orbitals are represented in red and blue, respectively. Auxiliary qubits employed to mediate the density-density interactions between α and β spin-orbitals are depicted in green. These layouts are provided as an example, as the simulation were carried out on two different QPU, and the qubits selection was performed prior each run to ensure reliable hardware partition.

The execution of the LUCJ run produces the `count_dict.txt`. This file stores the bitstrings corresponding to the quantum states generated through the run on the quantum hardware. Due to the noise inherent in current quantum hardware, the generated bitstrings include both physical and non-physical configurations. For this reason we employ a two step post-processing. The first post-processing step is performed by `run-sqd.py`, using the `qiskit-addon-sqd` 0.10.0 library. The noise perturbated configurations are filtered through

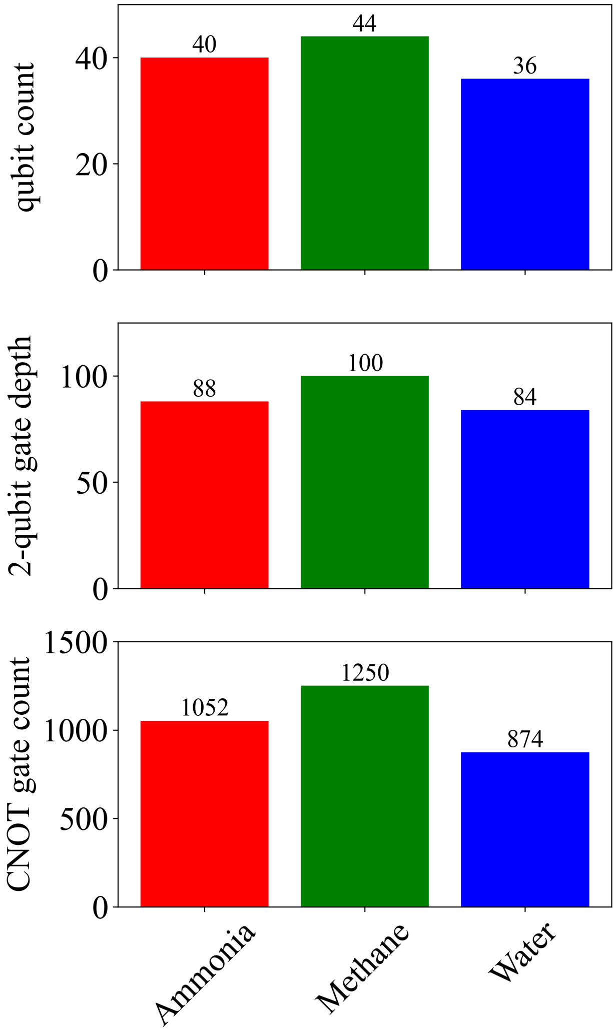

Figure S3: Qubit number, 2-qubits gate depth and CNOT gate count for the LUCJ circuit of (10e,20o) ammonia in red, (10e,22o) methane in green, and (10e,18o) water in blue.

a self-consistent configuration recovery (S-CORE) cycle that restores the correct number of particles and spin-z symmetries based on the distance from the current value of the given bit to the average occupancy of the spin-orbital.[10] The cleaned data is then divided into batches of configurations that span the effective subspace of the Hilbert space. For each batch the Hamiltonian of the active space is projected, diagonalized to retrieve the ground state. The resulting ground state is used to compute updated average orbital occupations. These occupations guide the probabilistic regeneration of new bitstrings, which in turn serve as input for the next S-CORE iteration. This procedure is repeated until the convergence is reached. Here, each sub-space is diagonalized using the external SBD solver, which is embedded in the workflow through the `solve_from_quick.py` file. As in the analogous HCI implementation, this file provides a customized CASCI kernel adapted for the SQD post-processing that receives the set of CI addresses, reconstructs the active-space integrals, writes AlphaDets.txt through `sbd_wrapper.py`. The execution of the SQD batches is controlled by `solver.py` through the SLURM submission template `submit_batch_template.sh`. For each batch, `solver.py` reconstructs the active space, reads the corresponding determinant file, and calls `solve_from_quick()`. The resulting SCI energies, SCI amplitudes, and orbital occupations are stored in `output_batch-*.npz`. Once the S-CORE is done, `convert_bitstr_matrix_to_address.py` transforms the bitstring representation of the selected batch into integer CI addresses. The ext-SQD refinement is applied only to the lowest-energy SQD batch and is done similarly to the SQD procedure. It is initiated by the `ext-SQD-run.py` that reconstructs the HF object with the active space uses the PyCI library (version 0.6.4) to build a wavefunction according to the d' defined in the config.yaml file. In addition, the ext-SQD subspace is augmented by generating all single excitations from each dominant determinant. The resulting set of determinants are written to `batchExtSQD-0.dat`. The ext-SQD diagonalization is executed by `solver_extSQD.py` via the SLURM template `submit_batch_template_extSQD.sh`. The solver file reads the `batchExtSQD-0.dat`, sets up the SBD temporary directory, and invokes the `solve_from_quick()` with gradient eval-

Table S1: Details of SQD calculations. For each system, we report the Active Space (AS); D_{AS} represents the Hilbert space dimension. $|\widetilde{\chi}_b|$ is the number of samples per batch. d represents the subspace (batch) with the lowest energy across all batches at the last iteration. The reported values for d correspond to the first step of the production run in aqueous environment.

**System**	**AS**	$\lvert\widetilde{\chi}_b\rvert$	$\boldsymbol{d}$	$\boldsymbol{D}_{\text{AS}}$
Ammonia	$(8e, 20o)$	1000	9.94×10^5	235.74×10^5
Methane	$(8, 22o)$	1000	13.41×10^5	535.09×10^5
Water	$(8e, 18o)$	1000	7.07×10^5	93.64×10^5

uation enabled. Finally, `ext-SQD-run.py` loads this archive, extracts the ext-SQD total energy and gradient, and generates the `QUICK_job_PySCF_modified.out`. The HF energy is extracted from the original `QUICK_job.out`, and the correlation energy is calculated as the difference between the ext-SQD and HF energies. The analytic gradient is only evaluated for the final ext-SQD subspace because only this iteration corresponds to the fully converged configuration-recovered CI space. This strategy significantly reduces computational overhead while providing fully analytic gradients for QM/MM molecular dynamics. In this work, all SQD and ext-SQD simulations were performed using the STO-3G basis, though both PySCF and the SQD/ext-SQD solvers support all-orbitals and frozen-core active-space options suitable for larger basis sets. All the SLURM jobs were created using the `submission_utilities.py`, as done for the HCI calculations. More details regarding the number of samples and configurations are reported in Table S1. In present paper the simulations performed with all of the orbitals within 6-31G* basis set. The STO-3G benchmark calculations discussed in the main text were performed using PySCF's native FCI solver and did not rely on the HCI workflow described here, which is used exclusively for the larger-basis 6-31G*

Benchmark of HCI and SQD/ext-SQD parameters

The parameters for both approaches were selected on a systematic benchmark against HCI single point reference energies, using methane as reference as it is the biggest system under

study. For the HCI calculations, a cutoff of $\epsilon_1 = 1 \cdot 10^{-5}$ was selected, as it differs only by 0.11 kcal/mol from the more stringent $\epsilon_1 = 1 \cdot 10^{-6}$, while reducing the computational cost to approximately 15 s per HCI batch using 24 CPUs. For the SQD and ext-SQD workflows, the selected configuration yields total energies within 2 kcal/mol of the HCI reference while sampling approximately 6 % of the full Hilbert space. The SQD and ext-SQD steps are applied sequentially, with the final energy evaluated by considering the combined SQD/ext-SQD workflow. In particular, an ext-SQD threshold of d' $= 1 \cdot 10^{-4}$ was employed, as it maintains the energy deviation within the 2 kcal/mol target while enabling an efficient refinement step with a wall-clock time of approximately 6 min. This setup was selected as a compromise between accuracy and post-processing efficiency, with each SQD batch requiring approximately 4 min on 24 CPU cores. Smaller sampled subspaces were found to lead to systematically larger deviations from the HCI reference energy.

Quantum-centric SQD simulations

Initially, a guess wavefuncion representing the ground state,$|\Phi_{qc}\rangle$, is extracted from the truncated version of the local unitary cluster Jastrow (LUCJ) ansatz[11] in the following form:

$$|\Phi_{\text{qc}}\rangle = e^{-\hat{K}_2} e^{\hat{K}_1} e^{i\hat{J}_1} e^{-\hat{K}_1} |x_{\text{RHF}}\rangle \tag{1}$$

here the one-body operators are denoted by $\hat{K}_1$ and $\hat{K}_2$, while $\hat{J}_1$ represents the density-density operator, and $|x_{\text{RHF}}\rangle$ is the restricted closed-shell HF state. The following fermions mapping on the physical qubits is performed with the Jordan-Wigner (JW) function. The amplitudes used to parametrize the LUCJ circuit are extracted from the gas-phase restricted closed-shell CCSD calculations within the selected active space, as done in previous quantum-centric studies.[10,12–15] The quantum state $|\Psi\rangle$ is sampled by executing the circuit over multiple shots in the computational basis. This procedure yields a collection of bitstrings, $\chi = \{\mathbf{x}_1 \ldots \mathbf{x}_d\}$, where each $\mathbf{x} \in \{0,1\}^M$ corresponds to a specific Slater determinant. These sampled configurations, distributed according to the empirical probability $\tilde{P}_\Psi$, constitute the basis for the subsequent algorithm stages. The fidelity of this distribution is inherently limited by the noise characteristic of current NISQ devices. This leads to the final bitstrings collection to have entries violating physical constraints, e.g. states with broken particle-number and spin-z symmetries. In this scenario, P_Ψ spreads over a Hilbert space containing configurations that do not contribute to the molecular ground states. This effect is mitigated through a configuration recovery protocol, which filters and refines the samples to restore the correct electronic determinants. For each configuration $\mathbf{x} \in \tilde{\chi}$ such that $N_\mathbf{x} \neq N$, a number of spin-orbitals is flipped according to the deviation between the measured state $x_{p\sigma}$ and the mean orbital occupancy $n_{p\sigma}$. The initial guess of the occupancies used in the first recovery iteration is computed from the raw quantum samples $\widetilde{\chi}$.[10] From the resulting refined set χ_R, we construct K many-body subspaces, $S(1), \ldots, S(K).$, by sampling batches of d con-

figurations according to their empirical frequencies. For each subspace, the Hamiltonian is projected and diagonalized as:

$$\hat{H}_{S^{(b)}} = \hat{P}_{S^{(b)}}\ \hat{H}\ \hat{P}_{S^{(b)}} \quad \text{with} \quad \hat{P}_{S^{(b)}} = \sum_{x\in S^{(b)}} |x\rangle\langle x| \tag{2}$$

The ground-state wavefunction $|\psi^{(b)}\rangle$ and its associated energy $E^{(b)}$ for each batch are determined using the iterative Davidson method. The best approximation of the ground-state energy is identified as the minimum value obtained across all batches, $min_b\ E^{(b)}$. To refine the process, the wavefunctions from all subspaces are used to update the average orbital occupancies:

$$n_{p\sigma} = \frac{1}{K}\sum_{b=1}^{K}\langle\psi^{(b)}|\hat{n}_{p\sigma}|\psi^{(b)}\rangle \tag{3}$$

These updated values are fed back into the configuration recovery step, initiating a self-consistent cycle. This iterative procedure continues until the energy and orbital occupancies reach numerical convergence.

Extended-SQD

After completing the SQD procedure and obtaining the subspace states $\{|\psi^{(b)}\rangle\}_{b=1}^{K}$ and their corresponding energies $E^{(b)}$, we perform a single ext-SQD correction step following the strategy presented by Barinson et al.[15,16] For the SQD batch with the lowest energy $S^{(b)} \subset \chi_R$, we first identify the dominant configurations based on their CI coefficients, using a threshold of $1\cdot 10^{-4}$. We then construct an enlarged configuration set by applying a selected set of fermionic single- excitation operator to these specific bitstrings in $S^{(b)}$. This subspace augmentation is carried out using the PyCI package.[17]

For any computational basis state $|x\rangle \in S^{(b)}$, the action of an excitation operator $\hat{E}_I$ maps the configuration to another computational basis state $|z_I\rangle$. The extended batch takes the form:

$$S_E^{(b)} = \{\ |x\rangle,\ \hat{E}_I|x\rangle\ \} \tag{4}$$

Ext-SQD assigns fully variational coefficients to all elements of $S_E^{(b)}$ and forms the Hamiltonian projected onto the extended space,

$$\hat{H}_{S_E^{(b)}} = \hat{P}_{S_E^{(b)}} \hat{H} \hat{P}_{S_E^{(b)}} \quad \text{with} \qquad \hat{P}_{S_E^{(b)}} = \sum_{z \in S_E^{(b)}} |z\rangle\langle z| \tag{5}$$

Diagonalization of $\hat{H}_{S_E^{(b)}}$ yields improved approximations to the ground and excited states within each batch. Only a single ext-SQD step is performed which allows to simultaneously enhance the variational flexibility of SQD while maintaining the moderate computational cost of these simulations.[16]

Book-end corrections values

Table S2 reports the classical HFE values, averaged over three replicates, together with the final values corrected using the STO-3G basis set. Table S3 reports only the corrections derived from HF/STO-3G, computed during the initial stages of the present work. Table S4 reports the corrections computed using HF/6-31G* for the same systems.

Table S2. HFE values obtained with MM approach and three book-ending correction protocols (all values are in kcal/mol).

**System**	**MM HFE**	**HF protocol**	**HF + FCI protocol**	**HF + SQD protocol**	**MNSol**
Ammonia	-2.64 ± 0.11	-2.79	-3.05	-3.20	-4.29
Methane	2.56 ± 0.04	2.41	1.72	2.19	2.00
Water	-9.12 ± 0.07	-3.45	-2.99	-3.33	-6.31

Table S3: Book-ending correction values computed with three different protocols. All values are expressed in kcal/mol.

**System**	**HF protocol**	**HF + FCI protocol**	**HF + SQD protocol**
Ammonia	-0.15 ± 0.47	-0.41 ± 0.12	-0.56 ± 0.24
Methane	-0.15 ± 0.07	-0.84 ± 0.33	-0.37 ± 0.90
Water	5.68 ± 0.30	6.13 ± 0.45	5.79 ± 0.30

Table S4: Book-ending correction values computed with three different protocols. All values are expressed in kcal/mol.

**System**	**HF protocol**	**HF + FCI protocol**	**HF + SQD protocol**
Ammonia	-3.69 ± 0.16	-3.67 ± 0.34	-3.29 ± 0.18
Methane	-0.47 ± 0.04	-0.45 ± 0.09	-0.40 ± 0.06
Water	-0.89 ± 0.39	-0.78 ± 0.33	-0.84 ± 0.26

Lennard-Jones re-parametrization

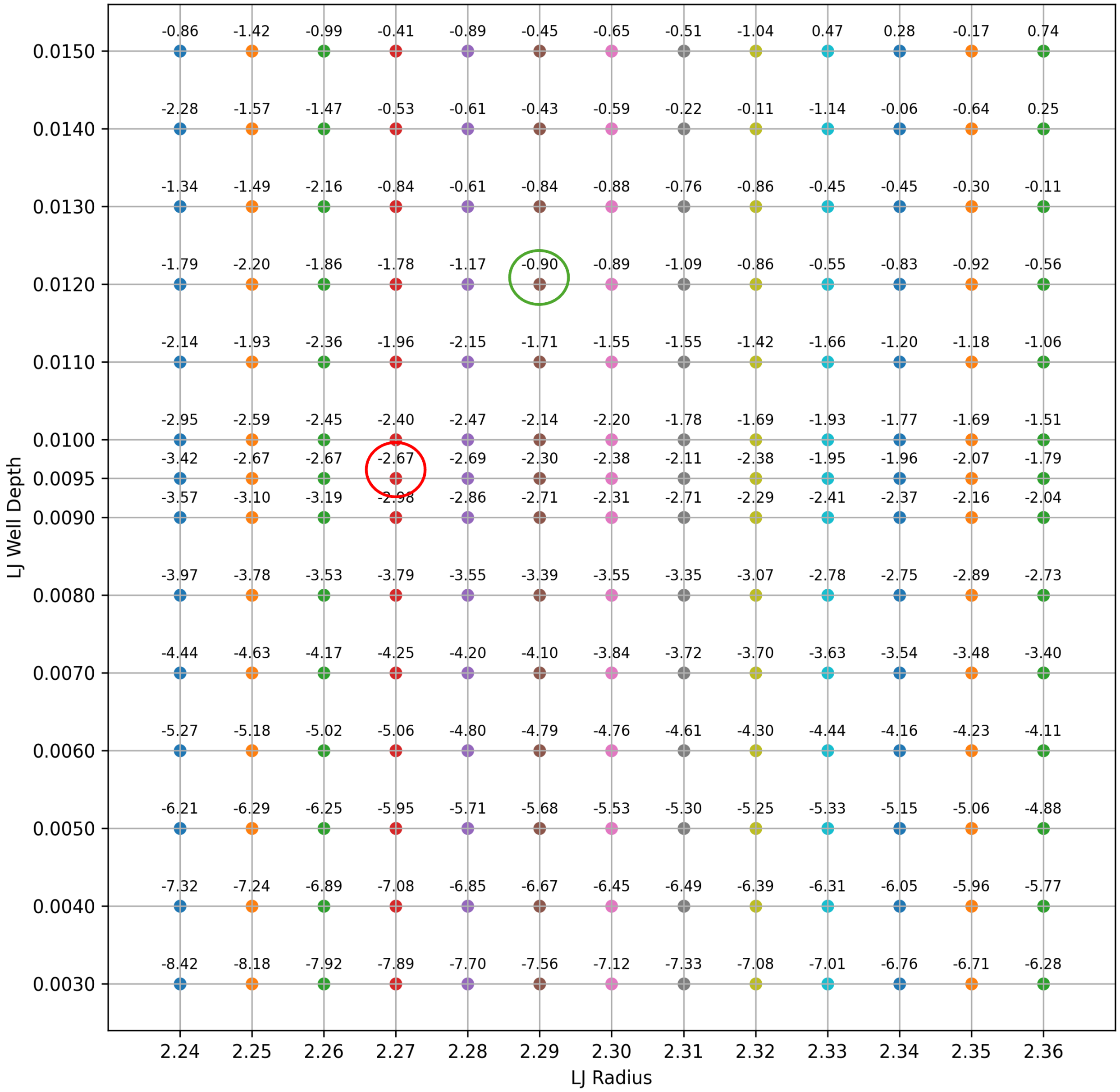

Figure S4: LJ re-parametrization grid for ammonia. Each point corresponds to the final HFE obtained for a given combination of LJ well depth (ε) and radius (σ). The red circle corresponds to the HFE obtained with the standard LJ parameters, while the green circle indicates the re-parametrized values selected for the present study.

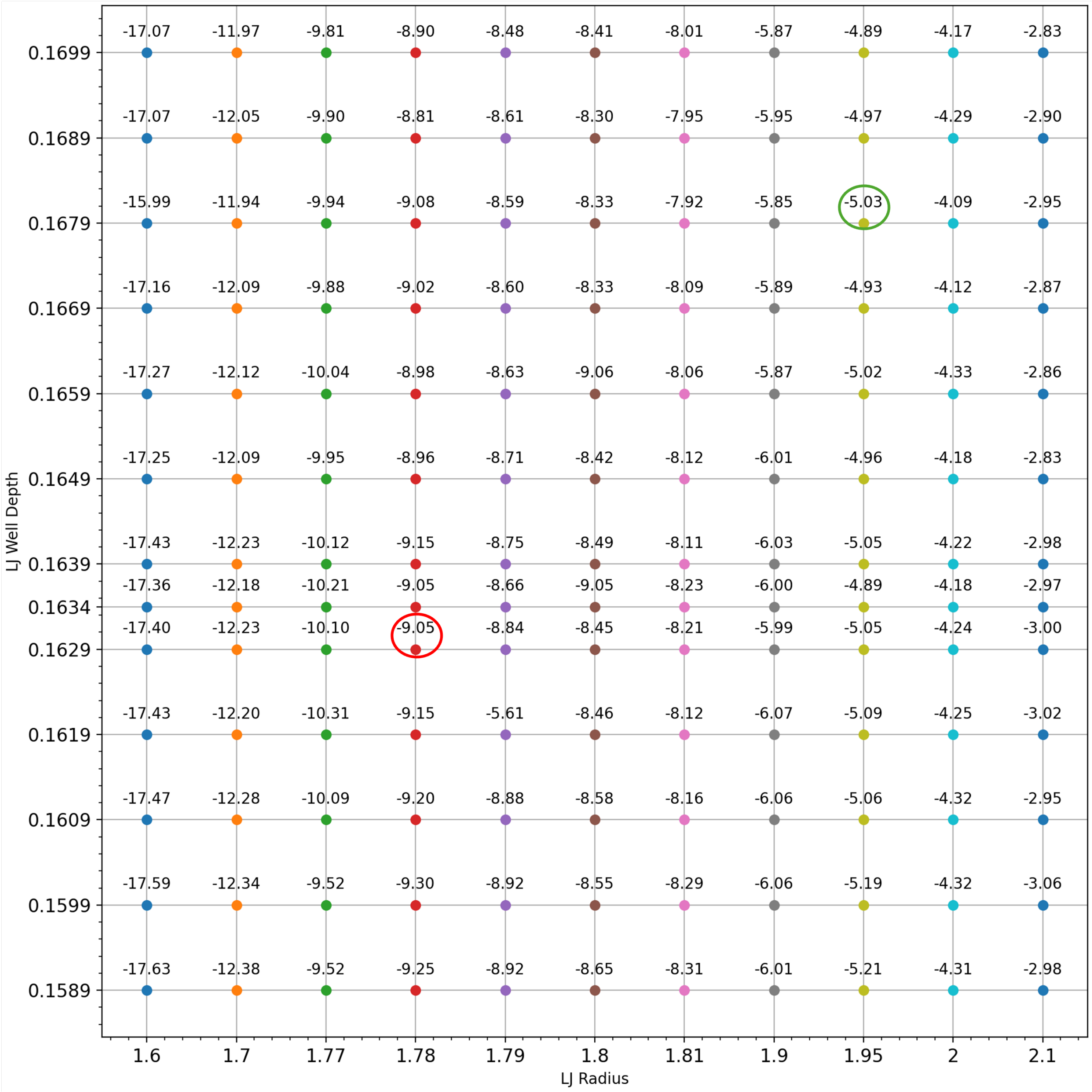

Figure S5: LJ re-parametrization grid for water. Each point corresponds to the final HFE obtained for a given combination of LJ well depth (ε) and radius (σ). The red circle corresponds to the HFE obtained with the standard LJ parameters, while the green circle indicates the re-parametrized values selected for the present study.

List of abbreviations used in this work

Abbreviation	Definition
AFE	Alchemical free energy
AFQMC	Auxiliary-field quantum Monte Carlo
AVAS	Atomic valence active space
BAR	Bennett acceptance ratio
CCSD	Coupled Cluster Singles and Doubles
CI	Configuration interaction
DFT	Density functional theory
DMET	Density matrix embedding theory
DMRG	Density matrix renormalization group
ext-SQD	Extended sample-based quantum diagonalization
FBI	File-based interface
FCI	Full configuration interaction
FCIQMC	Full configuration interaction quantum Monte Carlo
FEP	Free energy perturbation
FF	Force field
GAFF2	General AMBER force field 2
HCI	Heat-bath configuration interaction
HF	Hartree-Fock
HFE	Hydration free energy
LJ	Lennard-Jones
LUCJ	Local unitary cluster Jastrow
MBAR	Multistate Bennett acceptance ratio
MD	Molecular dynamics
MM	Molecular mechanics
MNSol	Minnesota solvation database
NISQ	Noisy intermediate-scale quantum
PBC	Periodic boundary conditions
QM	Quantum mechanics
QM/MM	Quantum mechanics/molecular mechanics
QPE	Quantum phase estimation
QPU	Quantum processing unit
RESP	Restrained electrostatic potential
SBD	Selected basis diagonalization
SCI	Selected configuration interaction
SQD	Sample-based quantum diagonalization
TI	Thermodynamic integration

References

(1) Manathunga, M.; Aktulga, H. M.; Götz, A. W.; Merz, K. M. Quantum Mechanics/Molecular Mechanics Simulations on NVIDIA and AMD Graphics Processing Units. *J. Chem. Inf. Model.* **2023**, *63*, 711–717.

(2) Cruzeiro, V. W. D.; Manathunga, M.; Merz, K. M.; Götz, A. W. Open-Source Multi-GPU-Accelerated QM/MM Simulations with AMBER and QUICK. *J. Chem. Inf. Model.* **2021**, *61*, 2109–2115.

(3) Case, D. A.; Aktulga, H. M.; Belfon, K.; Cerutti, D. S.; Cisneros, G. A.; Cruzeiro, V. W. D.; Forouzesh, N.; Giese, T. J.; Götz, A. W.; Gohlke, H. et al. AMBER24. 2024.

(4) Holmes, A. A.; Tubman, N. M.; Umrigar, C. Heat-bath configuration interaction: An efficient selected configuration interaction algorithm inspired by heat-bath sampling. *Journal of Chemical Theory and Computation* **2016**, *12*, 3674–3680.

(5) Sun, Q.; Berkelbach, T. C.; Blunt, N. S.; Booth, G. H.; Guo, S.; Li, Z.; Liu, J.; McClain, J. D.; Sayfutyarova, E. R.; Sharma, S. PySCF: the Python-based simulations of chemistry framework. *Wiley Interdisciplinary Reviews: Computational Molecular Science* **2018**, *8*, e1340.

(6) Sun, Q.; Zhang, X.; Banerjee, S.; Bao, P.; Barbry, M.; Blunt, N. S.; Bogdanov, N. A.; Booth, G. H.; Chen, J.; Cui, Z.-H. Recent developments in the PySCF program package. *The Journal of Chemical Physics* **2020**, *153*, 024109.

(7) Aleksandrowicz, G.; Alexander, T.; Barkoutsos, P. K.; Bello, L.; Ben-Haim, Y.; Bucher, D.; Cabrera-Hernández, F. J.; Carballo-Franquis, J.; Chen, A.; Chen, C.-F. et al. Qiskit: An Open-source Framework for Quantum Computing. 2019.

(8) Javadi-Abhari, A.; Treinish, M.; Krsulich, K.; Wood, C. J.; Lishman, J.; Gacon, J.;

Martiel, S.; Nation, P. D.; Bishop, L. S.; Cross, A. W. Quantum computing with Qiskit. *arXiv preprint arXiv:2405.08810* **2024**,

(9) ffsims developers ffsim: Faster simulations of fermionic quantum circuits. https://github.com/qiskit-community/ffsim, 2024; Accessed: 2024-09-01.

(10) Robledo-Moreno, J.; Motta, M.; Haas, H.; Javadi-Abhari, A.; Jurcevic, P.; Kirby, W.; Martiel, S.; Sharma, K.; Sharma, S.; Shirakawa, T. et al. Chemistry beyond the scale of exact diagonalization on a quantum-centric supercomputer. *Science Advances* **2025**, *11*, eadu9991.

(11) Motta, M.; Sung, K. J.; Whaley, K. B.; Head-Gordon, M.; Shee, J. Bridging physical intuition and hardware efficiency for correlated electronic states: the local unitary cluster Jastrow ansatz for electronic structure. *Chemical Science* **2023**, *14*, 11213–11227.

(12) Kaliakin, D.; Shajan, A.; Liang, F.; Robledo Moreno, J.; Li, Z.; Mitra, A.; Motta, M.; Johnson, C.; Ash Saki, A.; Das, S. et al. Accurate quantum-centric simulations of intermolecular interactions. *Communications Physics* **2025**, *8*, 396.

(13) Yu, J.; Moreno, J. R.; Iosue, J. T.; Bertels, L.; Claudino, D.; Fuller, B.; Groszkowski, P.; Humble, T. S.; Jurcevic, P.; Kirby, W. et al. Quantum-Centric Algorithm for Sample-Based Krylov Diagonalization. *arXiv preprint arXiv:2501.09702* **2025**, Submitted on 16 Jan 2025 (v1), last revised 24 Jan 2025 (this version, v2).

(14) Liepuoniute, I.; Doney, K. D.; Robledo Moreno, J.; Job, J. A.; Friend, W. S.; Jones, G. O. Quantum-Centric Computational Study of Methylene Singlet and Triplet States. *Journal of Chemical Theory and Computation* **2025**, *21*, 5062–5070.

(15) Barroca, M. A.; Gujarati, T.; Sharma, V.; Ferreira, R. N. B.; Na, Y.-H.; Giammona, M.; Mezzacapo, A.; Wunsch, B.; Steiner, M. Surface Reaction Simulations for Battery Ma-

terials through Sample-Based Quantum Diagonalization and Local Embedding. *arXiv preprint arXiv:2503.10923* **2025**, Submitted on 13 Mar 2025.

(16) Barison, S.; Robledo Moreno, J.; Motta, M. Quantum-centric computation of molecular excited states with extended sample-based quantum diagonalization. *Quantum Science and Technology* **2025**, *10*, 025034.

(17) Richer, M.; Sánchez-Díaz, G.; Martínez-González, M.; Chuiko, V.; Kim, T. D.; Tehrani, A.; Wang, S.; Gaikwad, P. B.; de Moura, C. E.; Masschelein, C. et al. PyCI: A Python-scriptable library for arbitrary determinant CI. *The Journal of Chemical Physics* **2024**, *161*.
